# Prediction of prognosis and immunotherapy response of tryptophan metabolism genes in acute myeloid leukemia

**DOI:** 10.3389/fphar.2025.1714246

**Published:** 2026-01-07

**Authors:** Tantan Zhong, Meirui Zhang, Xinran Qi, Zhihui Qi, Peipei Li, Na Wang, Xianghua Wang, Panpan Feng, Xiaosheng Fang

**Affiliations:** 1 Department of Hematology, Shandong Provincial Hospital Affiliated to Shandong First Medical University, Jinan, Shandong, China; 2 Department of Hematology, Shandong Provincial Hospital, Shandong University, Jinan, Shandong, China

**Keywords:** acute myeloid leukemia, fatty acid β-oxidation, immunotherapy, prognosis model, tryptophan metabolism

## Abstract

**Background:**

Acute myeloid leukemia (AML) is an aggressive and heterogeneous disease, associated with significant morbidity and mortality rates. Tryptophan metabolism has been implicated in the development of several tumors. The immune landscape within the tumor microenvironment plays a pivotal role in both leukemogenesis and the determination of patient prognosis. Nonetheless, the influence of tryptophan metabolic patterns and corresponding immune signatures in AML remains largely unclear.

**Methods:**

Transcriptomic, genomic, and clinical data from TCGA were analyzed, and GSE71014 was used for external validation. Molecular subtypes were identified via consensus clustering of tryptophan metabolism–related genes (TRPRGs). Immune infiltration was quantified using ESTIMATE. A tryptophan-related prognostic risk score (TRPRS) was constructed using LASSO–Cox regression and evaluated for prognostic performance.

**Results:**

We characterized alterations in 39 TRPRGs across AML cohorts and delineated the clinical and tumor microenvironmental features of two molecular subtypes. First, a TRPRG-based scoring system was established, identifying seven candidate genes significantly associated with patient outcomes. After LASSO-Cox regression selection, six genes were incorporated into the final prognostic model, stratify overall survival risk. The TRPRS effectively stratified overall survival in both the TCGA and GEO cohorts and remained an independent prognostic factor after multivariate adjustment. High-TRPRS patients exhibited distinct immune characteristics and differential drug sensitivity patterns. Functional experiments demonstrated that HADH and ECHS1 promote AML cell proliferation and survival.

**Discussion:**

Our integrative analysis identified key tryptophan-metabolism–related genes in AML and developed a six-gene TRPRS capable of accurately distinguishing survival risk. This model not only provides mechanistic insights into AML progression but also offers a framework for individualized risk stratification and therapeutic guidance.

## 1 Introduction

Acute myeloid leukemia (AML) is a biologically complex, molecularly and clinically heterogeneous disease. Over the past 5 years, advancements in treatment options for patients with newly diagnosed AML have led to improved outcomes for patients (DiNardo et al., 2023). Notable breakthroughs have occurred in AML immunotherapy and targeted therapy, particularly with the development of Fms Related Receptor Tyrosine Kinase 3 (FLT-3) inhibitors and Isocitrate Dehydrogenase (NADP (+)) 1/2 (IDH1/2) inhibitors (Kayser and Levis, 2022; Barrett, 2020). Despite the initial success in achieving remission, relapses remain a substantial challenge for AML. The prognostic evaluation of AML is complicated by its considerable molecular diversity and the highly variable composition of the tumor microenvironment (TME) (Menter and Tzankov, 2022), which also restricts accurate predictions of responses to immunotherapeutic interventions. Thus, the establishment of robust risk prediction models and the discovery of novel biomarkers are of paramount importance for accurate prognostic evaluation and the optimization of treatment strategies in AML. Cellular metabolism is fundamentally involved in the initiation and malignant progression of cancer. Emerging evidence indicates that glutamine, a semi-essential amino acid, supports tumor survival by contributing to biosynthetic pathways, energy generation, and the maintenance of redox balance (Zhang J. et al., 2017; Gong et al., 2022). Nevertheless, the molecular changes and metabolic characteristics of tryptophan in AML remain insufficiently characterized.

As an indispensable amino acid, tryptophan has a fundamental role in a diverse array of physiological functions. Its metabolites are critically involved in cell proliferation, maintenance of tissue homeostasis, and modulation of host responses to environmental cues and dietary inputs (Cervenka et al., 2017). For instance, tryptophan is required for protein synthesis and serves as a precursor to multiple bioactive metabolites involved in cell growth and homeostasis (Xue et al., 2023). Rather than functioning as part of a specific enzyme structure, tryptophan contributes to cellular processes by regulating protein translation and metabolic activity, thereby indirectly influencing cell proliferation. Accumulating studies suggest that tryptophan catabolism promotes to immune tolerance and modulates the efficacy of antitumor therapies primarily via the kynurenine pathway (Brochez et al., 2017). Within this metabolic route, the enzymes indoleamine 2,3-dioxygenase (IDO) and tryptophan 2,3-dioxygenase (TDO) mediate the first and biologically critical phase of tryptophan breakdown (Grohmann et al., 2017). Previous studies have demonstrated that IDO mediates immunosuppression (Munn et al., 1998), and its inhibition can restore antitumor immune responses (Yen et al., 2009). Likewise, TDO exhibits comparable immunosuppressive properties by suppressing T-cell proliferation and restricting immune cell infiltration (Opitz et al., 2011; Pilotte et al., 2012). Mechanistically, IDO-induced tryptophan depletion activates GCN2 while inhibiting mTOR signaling, ultimately impairing effector T-cell function (Giannone et al., 2020; Munn and Mellor, 2016). Metabolic pathways involving tryptophan have been widely implicated in the initiation and progression of multiple cancer types.

Recent research has elucidated the metabolic features of tryptophan in glioma, indicating that both oligodendrocytes and neurons can uptake tryptophan. Furthermore, increased concentrations of quinolinic acid, a byproduct of tryptophan catabolism, were increased in the cerebrospinal fluid, suggesting that tryptophan metabolism contributes to the pathophysiological mechanisms underlying glioma (Bosnyák et al., 2016). Collectively, these findings underscore the fundamental role of tryptophan metabolism in orchestrating neoplastic development and modulating anticancer immunity, highlighting its potential for informing immunotherapy strategies in AML.

Enoyl-CoA hydratase short chain-1 (ECHS1) and hydroxyacyl-CoA dehydrogenase (HADH) are mitochondrial enzymes that catalyze sequential steps in the β-oxidation of fatty acids and intersect with multiple amino acid metabolic pathways (Zhang Y. K. et al., 2017), including those of branched-chain amino acids and tryptophan. Both enzymes are essential for maintaining cellular energy homeostasis and metabolic flexibility, which are critical for the survival of rapidly proliferating tumor cells. ECHS1 facilitates the conversion of short-chain enoyl-CoA into 3-hydroxyacyl-CoA through hydration, while HADH oxidizes this product to yield 3-ketoacyl-CoA. These sequential reactions ultimately support acetyl-CoA generation, enabling its incorporation into the tricarboxylic acid cycle (Qu et al., 2020). Aberrant overexpression of ECHS1 and HADH has been reported in numerous tumors, associating with aggressive clinical behavior, poor prognosis, and increased metabolic adaptability (Du et al., 2021). Mechanistically, their upregulation may facilitate tumor progression by enhancing amino acid catabolic flux. thereby supporting biosynthetic pathways and maintaining redox homeostasis (Li et al., 2025). The inhibition of the expression of either gene could impair tumor cell proliferation, induce apoptosis, and alter the gene expression level of tryptophan metabolism, indicating that ECHS1- and HADH-driven amino acid metabolism may contribute to tumor development. However, the precise mechanisms linking these enzymes to amino acid metabolism and their role in tumor progression in AML remain to be elucidated.

In this study, we comprehensively analyzed the transcriptomic and clinical data from The Cancer Genome Atlas (TCGA) and Gene Expression Omnibus (GEO) databases to investigate the expression patterns, prognostic significance, and immunological implications of TRPRGs in AML. Using unsupervised consensus clustering, we classified patients into distinct molecular subtypes, which displayed significant differences in amino acid metabolism, biological pathways, and tumor immune microenvironment features. Subsequently, we established a prognostic risk model derived from TRPRGs through Least Absolute Shrinkage and Selection Operator (LASSO)—Cox regression and validated its predictive efficacy across independent GEO datasets. Additionally, we performed functional assays in AML cell lines to confirm that the knockdown of ECHS1 or HADH suppresses leukemia cell proliferation, and induces apoptosis providing mechanistic insight into the metabolic and immunological roles of tryptophan and presenting a potential framework for improved risk stratification and novel therapeutic targets in AML.

## 2 Materials and methods

### 2.1 Data collection

Transcriptomic data and associated clinico-pathological information were retrospectively acquired from TCGA, encompassing 151 individuals diagnosed with AML. For comparative purposes, 337 peripheral blood specimens from healthy donors were obtained from the Genotype-Tissue Expression (GTEx) project to serve as non-malignant controls. Cases lacking complete clinical annotations were excluded from further analysis. Furthermore, transcriptomic profiles and clinical information from 104 AML patients (accession number GSE71014, platform GPL10558) and 140 AML patients (accession number GSE37642, platform GPL570) within the GEO repository were included as external validation sets to evaluate the stability of the prognostic signature. For subsequent analytical steps, 40 genes associated with tryptophan metabolism were obtained from the Molecular Signatures Database (MSigDB, release 7.4).

Gene expression data from the TCGA-LAML and GTEx cohorts were generated using RNA sequencing (RNA-seq) and normalized to transcripts per million (TPM) prior to analysis. The GSE71014 dataset was derived from a microarray platform (GPL10558) and normalized using the Robust Multi-array Average method. To ensure cross-cohort comparability, all data were log_2_-transformed, and potential batch effects were corrected using the ComBat function of the R package “sva” before downstream analyses.

### 2.2 Consensus molecular clustering based on TRPRGs

In this study, we selected seven tryptophan metabolism-associated genes with prognostic relevance. Using the “ConsensusClusterPlus” package, consensus clustering was performed with 50 iterations, employing the following parameters: pItem = 0.8, pFeature = 1, and clusterAlg = “K-Means.” The optimal cluster count was determined by assessing the cumulative distribution function plots and consensus heatmaps, which indicated a final K value of 2. Subsequently, the “survival” package was used to compare differences in survival outcomes among AML molecular subtypes derived from TRPRGs. Principal component analysis (PCA) was conducted using the “ggplot2” R package to visualize the spatial arrangement of molecular subgroups defined by TRPRGs. The expression patterns of the seven TRPRGs across subgroups were displayed using pheatmap. Pathway-level differences among tryptophan metabolism subtypes were evaluated through Gene Set Variation Analysis (GSVA) with reference to gene sets obtained from the “c2.cp.kegg_legacy.v2023.2.Hs.symbols” collection in MsigDB.

### 2.3 Characterization of the immune landscape in AML

Immune cell infiltration in the TCGA-derived high- and low-TRPRS subgroups was assessed using the Estimation of STromal and Immune cells in MAlignant Tumor tissues using Expression data (ESTIMATE) algorithm. To further characterize the immune landscape, the CIBERSORT (Cell-type Identification By Estimating Relative Subsets Of RNA Transcripts) method was applied to TPM-normalized TCGA-LAML transcriptome data to evaluate the relative proportions of 22 distinct immune cell populations. Samples yielding a CIBERSORT output with p > 0.05 were excluded, and only the remaining qualified cases were retained for subsequent analyses (Newman et al., 2015). To characterize immune infiltration in the AML TME, we applied the single-sample gene set enrichment analysis (ssGSEA) algorithm, employing curated gene signatures for each immune cell subset derived from current literature (Ru et al., 2019; He et al., 2018).

### 2.4 Construction and assessment of a prognostic model based on TRPRGs

Univariate Cox regression was conducted to determine the prognostic significance of tryptophan metabolism-associated genes in AML, with a threshold of p < 0.05 used to screen for genes exhibiting a significant correlation with overall survival. Subsequently, differentially expressed genes identified across subgroups were analyzed using LASSO regression to facilitate dimension reduction and mitigate model overfitting. A stepwise multivariate Cox regression analysis was subsequently performed to identify independent prognostic factors, forming the basis for constructing the tryptophan-related prognostic risk score (TRPRS) in AML (Gui and Li, 2005). The individual TRPRS for each patient was calculated based on a weighted linear combination of the expression values of prognostic TRPRGs, using the corresponding coefficients derived from multivariate Cox regression, as defined by the following algorithm: TRPRS = 0.235 × ACAT2 + 0.237 × ECHS1 + 0.201 × HADH +0.300 × IL4I1 + 0.380 × KMO + 0.499 × MAOA. The TCGA dataset served as the training cohort, and the GSE71014 dataset obtained from the GEO repository was used for independent validation. The classification of the AML cohort into low- and high-TRPRS strata was determined by applying the median TRPRS as a cutoff. Survival outcomes across defined subgroups were assessed using Kaplan–Meier curves, with statistical differences evaluated using the log-rank test. Time-dependent receiver operating characteristic (ROC) curve analysis was conducted in the validation set to evaluate the model’s predictive ability. Area under the curve (AUC) values were derived to estimate the discriminative power for one-, three-, and five-year overall survival probabilities.

### 2.5 Analysis of genomic alterations

Somatic mutation and copy number alteration (CNA) profiles for the AML cohort were acquired from TCGA database (https://portal.gdc.cancer.gov/). Subsequent visualization and examination of mutational and CNV landscapes were performed using the “maftools” package in the R (Mayakonda et al., 2018).

### 2.6 Integrated evaluation of six prognostic models

Initially, the mRNA expression of ACAT2, ECHS1, HADH, IL4I1, KMO, and MAOA was evaluated using data from the TCGA and GEO repositories, comparing levels between AML patients and normal controls. Based on the median expression of these genes, AML patients were stratified into high- and low-expression groups, after which Kaplan-Meier analysis was performed to compare survival outcomes between these subgroups.

### 2.7 Drug response profiling

Drug response data were sourced from the Genomics of Drug Sensitivity in Cancer database (GDSC2, https://www.cancerrxgene.org/) by retrieving gene expression profiles and associated drug sensitivity measurements. Ridge regression algorithms were employed to develop predictive models suitable for AML, using sensitivity data covering a panel of 198 compounds. Drug sensitivity predictions for the TCGA-LAML cohort were subsequently derived using the “oncoPredict” package in R.

### 2.8 Culture and transfection of cells with siRNA

AML cell lines (MOLM-13 and MV4-11) were maintained until reaching the logarithmic growth phase. Cells were cultured in Iscove’s Modified Dulbecco’s Medium (Gibco, United States) supplemented with 10% fetal bovine serum (Gibco, United States) and 1% penicillin–streptomycin at 37 °C in a 5% CO_2_ humidified incubator.

Upon reaching approximately 80% confluence in 6-well plates, cells were prepared for transfection. First, 50 nM siRNA targeting HADH or ECHS1 (designed and synthesized by General Bio, Anhui, China) was diluted in 125 µL of Opti-MEM I Reduced Serum Medium (serum-free; Thermo Fisher Scientific, United States). Additionally, 5 µL of Lipofectamine 3000 transfection reagent (Invitrogen, CA, United States) was mixed with an equivalent volume of Opti-MEM I Reduced Serum Medium (125 µL). Both solutions were incubated at room temperature for 5 min. Subsequently, the two mixtures were gently combined to a total volume of 250 μL and incubated for 15 min to enable the formation of siRNA-lipid complexes suitable for transfection. Following complex formation, a total of 250 μL of the siRNA-lipid mixture was carefully dispensed into each well of a 6-well plate that had been preloaded with 2 mL of serum-free Opti-MEM medium. To facilitate uniform adherence, culture plates were gently swirled in cross-shaped or diagonal patterns. Cells were cultured for 12 h at 37 °C under a humidified 5% CO_2_ atmosphere, after which the transfection medium was aspirated and substituted with complete growth medium. The cells were then returned to the incubator and allowed to grow for an additional 24–48 h under standard conditions before further analysis.

To ensure experimental rigor, control groups were included for all transfection assays: a non-targeting siRNA (si-NC) served as a negative control under identical conditions, and a mock group (Lipofectamine reagent only, without siRNA) was included to account for any transfection-related cytotoxicity. All experiments were independently repeated three times, after which cells were collected 48 h post-transfection for RNA and protein extraction to evaluate knockdown efficiency through RT-qPCR and Western blotting.

### 2.9 Extraction of total RNA followed by cDNA synthesis

Total RNA was extracted via TRNzol reagent (TIANGEN, China). Briefly, 1 mL of TRNzol was added to each well of cultured cells and allowed to incubate at room temperature for 5 min following complete lysis. Subsequently, 0.2 mL of chloroform was added per 1 mL of TRNzol reagent, with thorough vortexing for 15 s. Each sample was then allowed to remain undisturbed at room temperature for 3 min to promote phase separation. Centrifugation was performed at 12,000 × g for 15 min at 4 °C. The aqueous layer was carefully transferred to a fresh tube, mixed with an equal volume of isopropanol, and incubated at room temperature for 10 min before a second round of centrifugation to pellet the RNA. Following a wash with 75% ethanol, the precipitate was air-dried to evaporate residual solvent and dissolved in RNase-free ddH_2_O for subsequent use. Complementary DNA (cDNA) was synthesized using the SweScript All-in-One RT SuperMix for qPCR kit (Servicebio, China) according to the manufacturer’s protocol.

### 2.10 Quantitative real-time PCR

Quantitative real-time PCR (qPCR) was conducted using the SuperReal PreMix Plus kit containing SYBR Green (TIANGEN, China) on a real-time PCR detection system. Each 20 μL qPCR system comprised of 10 μL of 2 × SYBR Green Master Mix, 0.6 μL each of forward and reverse primers (10 μM), 1 μL cDNA template, and nuclease-free water supplemented to achieve the final volume. Amplification was performed under the following protocol: initial denaturation at 95 °C for 15 min; 40 cycles of denaturation at 95 °C for 10 s, followed by annealing/extension at 60 °C for 30 s. Each sample was run in three technical replicates to ensure experimental consistency. The Relative gene expression levels were determined via the 2^−ΔΔCt method. The primer sequences employed include: HADH, forward: GTG​ACG​CAT​CCA​AAG​AAG​ACA​TT, reverse: TGG​GTT​CTC​TGC​ATC​CAT​TTC​AT; ECHS1, forward: CCT​CGG​GTG​CTA​ACT​TTG​AGT​AC, reverse: TGG​GTT​CTC​TGC​ATC​CAT​TTC​AT.

### 2.11 Assessment of cell proliferation

Cells were seeded into 96-well plates at a density of 5 × 10^4^ cells per milliliter, with 100 µL of suspension added to each well. Following incubation for 24, 48, and 72 h, 10 µL of CCK-8 solution (Meilunbio, China) was added to every well. Plates were maintained at 37 °C for 2–4 h, after which absorbance at 450 nm was measured using a microplate reader (Molecular Devices, CMax Plus, United States).

### 2.12 Evaluation of apoptosis

Apoptosis was assessed using an Annexin V-FITC/propidium iodide (PI) Apoptosis Detection Kit (Lianke Bio, AP101-01, China). Both attached and suspended cells were collected, rinsed twice with chilled PBS, and re-suspended in 1× Binding Buffer. Approximately 1–10 × 10^5^ cells were stained with 5 µL of Annexin V-FITC and 10 µL of PI, followed by incubation for 5 min at room temperature in the dark. Samples were analyzed immediately using a FACSVerse flow cytometer (CytoFLEX, BECKMAN, United States).

### 2.13 Statistical analysis

Statistical analysis and plotting were performed using R (version 4.3.3). The Wilcoxon test was employed to compare two paired groups, while categorical variables were assessed using the Chi-square test or Fisher’s exact test. For analyses involving multiple comparisons, including immune cell infiltration quantification (CIBERSORT, ssGSEA) and differential expression of immune-related genes, p-values were adjusted using Benjamini–Hochberg false discovery rate (FDR) correction. Adjusted p-values (FDR) < 0.05 were considered statistically significant for these analyses. In contrast, GSVA was employed as an exploratory tool to characterize biological pathways between pre-defined subtypes, with results presented through nominal p-values. Differences in survival were determined by the log-rank test. Statistical significant was defined as *p < 0.05, **p < 0.01, and ***p < 0.001. All statistical tests were two-sided.

## 3 Results

### 3.1 Genomic alterations and expression profiles of TRPRGs in AML

**FIGURE 1:**
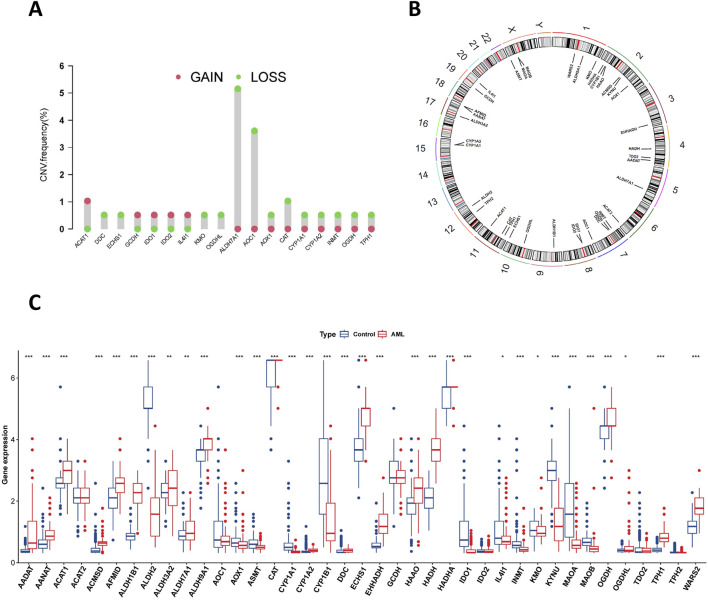
Genetic variations and transcriptional expression of TRPRGs in AML. **(A)** Frequencies of CNV alterations of TRPRGs in AML. The height of the column represents the alteration frequency. **(B)** Chromosomal locations of TRPRG CNV alterations. **(C)** Expression distributions of 39 TRPRGs between AML and normal controls from GTEx. *p < 0.05, **p < 0.01, ***p < 0.001.

This study focused on 39 TRPRGs. Among AML patients, genomic alterations in these TRPRGs were observed in 3.23% (4/124) of cases (Supplementary Figure S1). The overall low mutation frequency of TRPRGs suggests that their dysregulation in AML is primarily driven by transcriptional or post-transcriptional mechanisms rather than somatic mutations. Among these, MAOA exhibited the highest mutation frequency, followed by OGDHL and TPH1. Further analysis of somatic CNA among tryptophan-related genes revealed frequent genomic changes within the 39 TRPRPs. Notably, ACAT1, GCDH, IDO1/2, and IL4I1 exhibited widespread copy number amplifications, while numerous genes, including ALDH7A1, AOC1, DDC, ECHS1, and CAT, presented with copy number deletions (Figure 1A). Figure 1B depicts the chromosomal distribution of copy number variations in tryptophan metabolism—related genes. We further investigated the differential expression level of tryptophan metabolism-related genes were evaluated between AML tumors and normal tissues. The results revealed that multiple TRPRGs exhibited higher expression in tumor tissues, such as ECHS1 and HADH (Figure 1C).

### 3.2 Identification of tryptophan metabolism subtypes in AML

**FIGURE 2:**
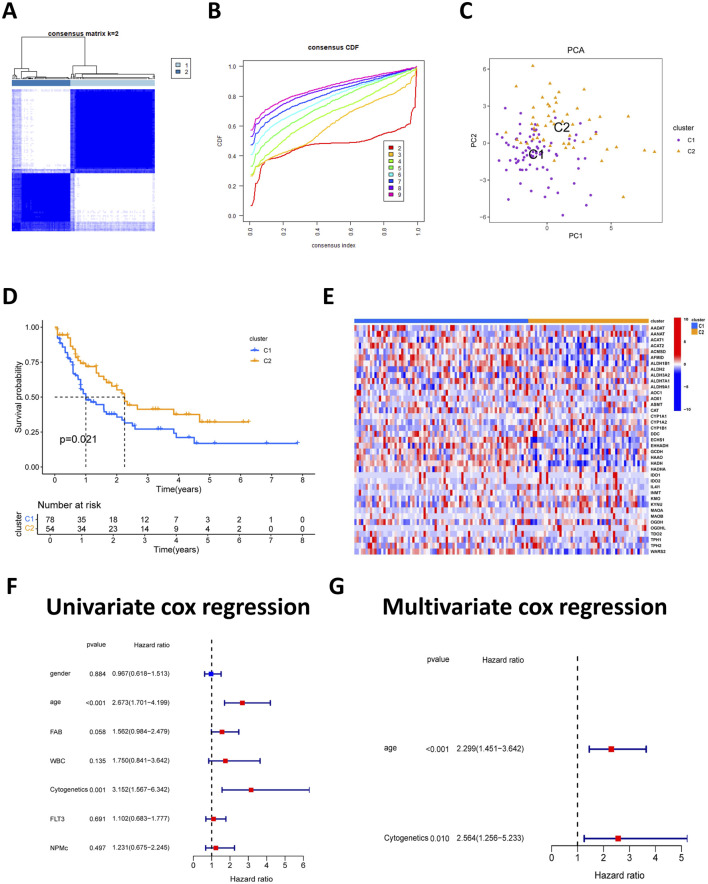
Subtype classification of TRPRGs based on consensus clustering. **(A)** Consensus matrix heatmap and correlation areas for two clusters (k = 2). **(B)** Cumulative distribution function analysis showing optimal clustering at k = 2. **(C)** PCA plot illustrating significant separation between the two clusters. **(D)** Kaplan–Meier survival analysis indicating improved prognosis in C2. **(E)** Differential expression of TRPRGs between the two subtypes. **(F)** Univariate cox regression analysis of prognostic factors and TRP subtypes. **(G)** Multivariate cox regression analysis of prognostic factors and TRP subtypes.

Univariate Cox regression analysis identified seven TRPRGs significantly associated with overall survival in AML patients, as illustrated by the corresponding forest plot. Consensus clustering based on the expression of these seven TRPRGs was performed to stratify AML patients and explore subtype-specific molecular and clinical features. The optimal cluster number (k-value) was identified as k = 2 following calculation of the consensus index. Consequently, the cohort was divided into two molecular subtypes, designated C1 (n = 78) and C2 (n = 54) (Figures 2A,B). PCA confirmed a balanced distribution of AML patients across both clusters (Figure 2C). Kaplan—Meier analysis demonstrated that patients in subtype C2 had significantly longer overall survival than those in C1 (p < 0.05) (Figure 2D). Expression profiling revealed that several TRPRGs, including AANAT, KMO, and MAOA, were more highly expressed in subtype C2, while ECHS1, HADH, and ACAT2 exhibited increased expression in subtype C1 (Figure 2E). Univariate and multivariate Cox regression analyses further validated the C1/C2 subtype as an independent prognostic factor in AML (Figures 2F,G). To characterize the biological differences between these subtypes, functional enrichment analysis was performed. GSVA revealed that subtype C1 was enriched in various amino-acid-related metabolic pathways, reflecting distinct metabolic programs between C1 and C2 (Supplementary Figure S2).

### 3.3 Tumor microenvironment features between the C1 and C2 molecular subtypes

**FIGURE 3:**
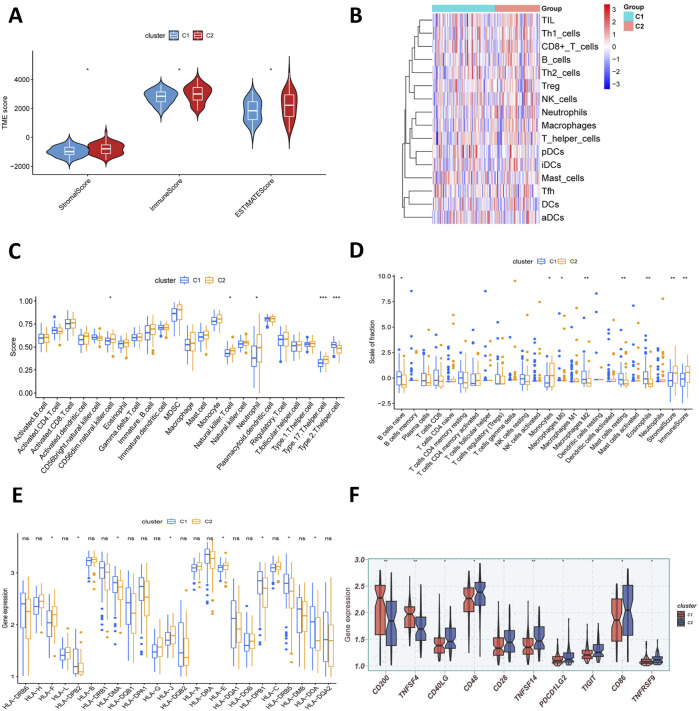
Immune microenvironment of TRPRG-based molecular subtypes. **(A)** Immune score, ESTIMATE score, and stromal score quantifying immune differences between subtypes. **(B)** Activity of immune-related pathways in C1 versus C2. **(C,D)** Abundance of TME-infiltrating cell types estimated using CIBERSORT and ssGSEA in different subtypes. **(E)** Differential expression of HLA molecules in C1 and C2. **(F)** Differential expression of various immune checkpoints in C1 and C2.

To investigate TME characteristics associated with tryptophan metabolism, the ESTIMATE algorithm was applied to compare stromal and immune infiltration between C1 and C2 molecular subtypes. Subtype C2 demonstrated higher stromal and immune scores compared to subtype C1 (Figure 3A). Furthermore, immune cell populations and their functional pathways were assessed via ssGSEA, revealing no significant differences in immune-related pathway activity between the two subgroups (Figure 3B). Using the CIBERSORT method, our analysis identified increased abundance of plasma cells, CD8^+^ T cells, and M2 macrophages in the C1 subgroup, whereas the C2 subgroup showed elevated infiltration levels of CD4^+^ T cells (Figure 3D).

We then employed the ssGSEA method to assess the relationship of the two clusters with a panel of 23 immune cell types. The findings demonstrated that the C2 subgroup exhibited significantly higher infiltration of MDSCs, neutrophils, immature B cells, and natural killer (NK) T cells, whereas the C1 subgroup showed elevated levels of activated CD4^+^ T cells, CD56^brigh NK cells, and memory B cells (Figure 3C). Additionally, the C1 subgroup demonstrated pronounced upregulation of antigen presentation—associated genes indicating distinct immune functional characteristics. Further analyses of immune checkpoint expressions revealed significant differential expressions of multiple critical molecules, such as CD200, PDCD1LG2, TNFSF4 (OX40L), and CD40LG (CD40L), suggesting divergent immunoregulatory profiles across the subgroups. These findings imply that tryptophan metabolism-related signatures are crucial in immunotherapy (Figures 3E,F).

The TIDE (Tumor Immune Dysfunction and Exclusion) algorithm was employed to compare immune escape scores between subgroups, indicating a higher score in C1, suggesting increased immune evasion in this subtype. However, no statistically significant difference was observed between the two subtypes (Supplementary Figure S3). Based on survival-associated tryptophan metabolism genes, 132 AML patients were stratified into two molecular subtypes. Functional and immune profiling revealed substantial differences, with C2 displayed higher stromal and immune scores and pronounced activation of immune-related pathways, reflecting a complex balance of antitumor immunity and immunosuppression. Conversely, C1 was marked by poorer survival outcomes, activation of oncogenic and proliferative pathways, increased CD8^+^ T-cell infiltration, and features suggestive of immune escape, highlighting potential implications for prognosis and therapeutic targeting.

### 3.4 Comparison of drug sensitivity between the two subgroups

**FIGURE 4:**
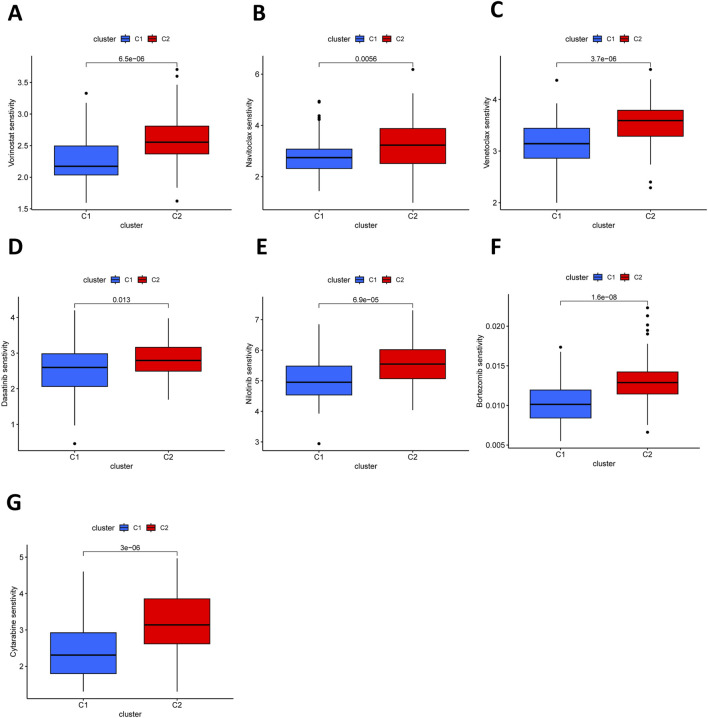
Drug sensitivity analyses in two subtypes. Vorinostat **(A)**, Navitoclax **(B)**, Venetoclax **(C)**, Dasatinib **(D)**, Nilotinib **(E)**, Bosutinib **(F)**, and Cytarabine **(G)** were observed a more sensitive response in the C2 than in the C1.

Chemotherapy and targeted therapy are vital strategies in managing patients with AML. Therefore, it is essential to investigate differences in drug sensitivity between the identified subgroups. Standard cytotoxic agents, including histone deacetylase inhibitors such as vorinostat, demonstrated greater sensitivity in the C2 subgroup (Figure 4A). BCL-2 inhibitors navitoclax (Figure 4B) and venetoclax (Figure 4C) enhance chemotherapy and improve efficacy in patients with AML with an fms-like tyrosine kinase (FLT3) mutation (Stone et al., 2017). Nilotinib, dasatinib, and bosutinib, as allosteric inhibitors of BCR-ABL, exhibited higher sensitivity in the C2 subgroup (Figures 4D–F). Furthermore, a more sensitive response to cytarabine (Ara-C), a first-line treatment drug for AML, was noted in the C2 compared to the C1 subgroup (Figure 4G). These results indicated that the two subgroups exhibit markedly divergent responses to chemotherapy and targeted therapy.

### 3.5 Development and validation of TRPRS

**FIGURE 5:**
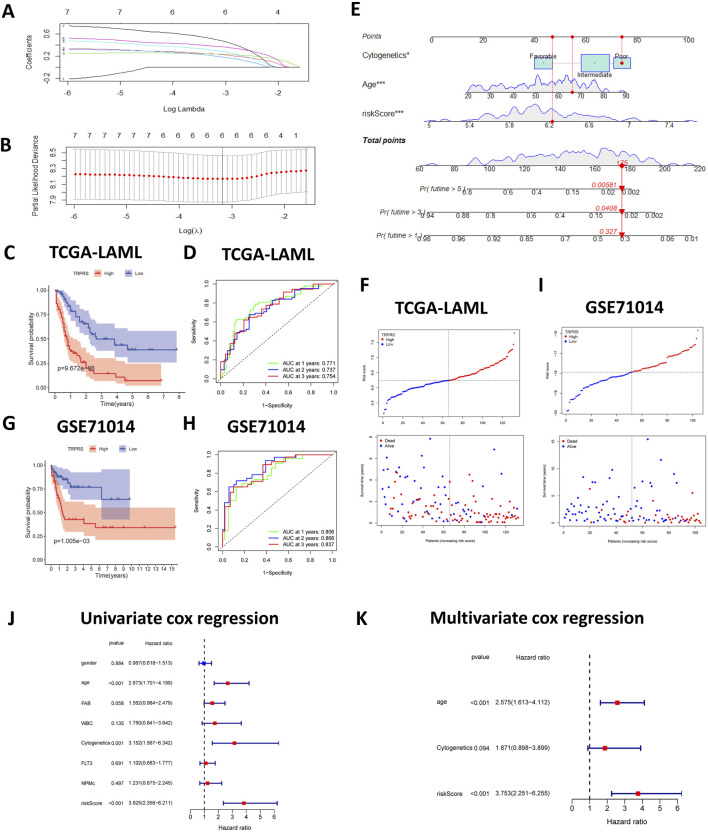
Construction and validation of TRP-related prognostic features. **(A)** Ten-fold cross-validation of parameter selection adjusted by LASSO regression. **(B)** LASSO coefficient profiles with a vertical line at the optimal value determined by cross-validation. **(C)** Kaplan-Meier curves comparing high- and low-TRPRS groups in TCGA-LAML. **(D)** Time-dependent ROC curve analysis in the TCGA-AML cohort. **(E)** Nomogram combining cytogenetics and age, and TRPRS. **(F)** Distribution of TRPRS values and patient survival between low and high-TRPRS groups in the TCGA-LAML. **(G)** KM curves demonstrating survival status between high and low-TRPRS groups for GSE71014. **(H)** Time-dependent ROC curve analysis in the GSE71014 cohort. **(I)** TRPRS distribution and survival status in GSE71014 patients. **(J)** Univariate and **(K)** multivariate COX regression analyses for prognostic factors and different clinical features. *p < 0.05. **p < 0.01. ***p < 0.001.

Prognostic genes were identified in the TCGA-LAML training cohort to construct a predictive model. Univariate Cox regression stratified patients into high- and low-TRPRS groups using optimal gene expression cutoffs. We identified seven TRPRGs significantly associated with reduced overall survival in AML patients. A 10-fold cross-validated LASSO regression was subsequently applied to these genes, leading to the selection of six key TRPRGs (ACAT2, ECHS1, HADH, IL4I1, KMO, and MAOA) for model construction. The TRPRS for each patient was calculated using the formula: TRPRS = 0.235 × ACAT2 + 0.237 × ECHS1 + 0.201 × HADH + 0.300 × IL4I1 + 0.380 × KMO + 0.499 × MAOA (Figures 5A,B). Patients were stratified into high-TRPRS and low-TRPRS groups based on the median TRPRS value. Kaplan-Meier curves demonstrated that the high-TRPRS group exhibited significantly poorer overall survival (*p <* 0.01; Figures 5C,G). Additionally, ROC curve analysis evaluated the predictive accuracy of the TRPRS, yielding AUC values of 0.771, 0.737, and 0.754 at 1, 3, and 5 years for the TCGA training cohort, respectively, supporting the model’s ability to reliably stratify AML patients according to survival risk. For the GSE71014 cohort, AUC values were 0.806, 0.866, and 0.837 at 1, 3, and 5 years, respectively (Figures 5D,H). To further strengthen the external validation of the TRPRS, we evaluated its prognostic performance in an additional GEO cohort (GSE37642). Consistent with the findings from TCGA-LAML and GSE71014, the TRPRS effectively stratified patients in GSE37642 into high- and low-TRPRS groups with significantly different overall survival outcomes (p < 0.05). ROC curve analysis also demonstrated stable predictive performance at 1, 3, and 5 years in this cohort (Supplementary Figure S4). These results confirm the reproducibility and generalizability of the TRPRS across independent datasets. Although the GSE71014 cohort included 104 AML samples with available gene expression and survival data, it lacked detailed clinical annotations such as age, sex, and cytogenetic risk, limiting direct baseline comparison with the TCGA-LAML training cohort. Nevertheless, the TRPRS maintained strong predictive performance across this independent dataset, indicating the robustness and generalizability of the model. Furthermore, we evaluated the predictive performance of our model against two previously established metabolism-associated prognostic signatures. Cao et al. constructed a prognostic signature based on NAD metabolism in AML, which yielded time-dependent AUC values of 0.69, 0.68, and 0.69 for 1-, 2-, and 3-year overall survival, respectively. Conversely, Ren et al. established a model focusing on metabolic genes associated with immunotherapy response in AML, reporting AUCs of 0.706, 0.690, and 0.710 for 1-, 3-, and 5-year survival within the TARGET cohort. In comparison, our TRPRS demonstrated superior predictive accuracy for AML patient outcomes.

To enhance the clinical utility of the TRPRS, a prognostic nomogram integrating this signature was developed (Figure 5E). Calibration curves indicated close agreement between nomogram predictions and actual observed survival outcomes (Supplementary Figure S5). A heatmap was generated to visualize the distribution of TRPRS values and patient survival status, revealing increased mortality and distinct TRPRG expression patterns in high-TRPRS patients (Figures 5F,I). Univariate and multivariate Cox analyses confirmed that TRPRS is an independent prognostic predictor in AML (Figures 5J,K).

### 3.6 TME and immune checkpoints in TRPRSs

**FIGURE 6:**
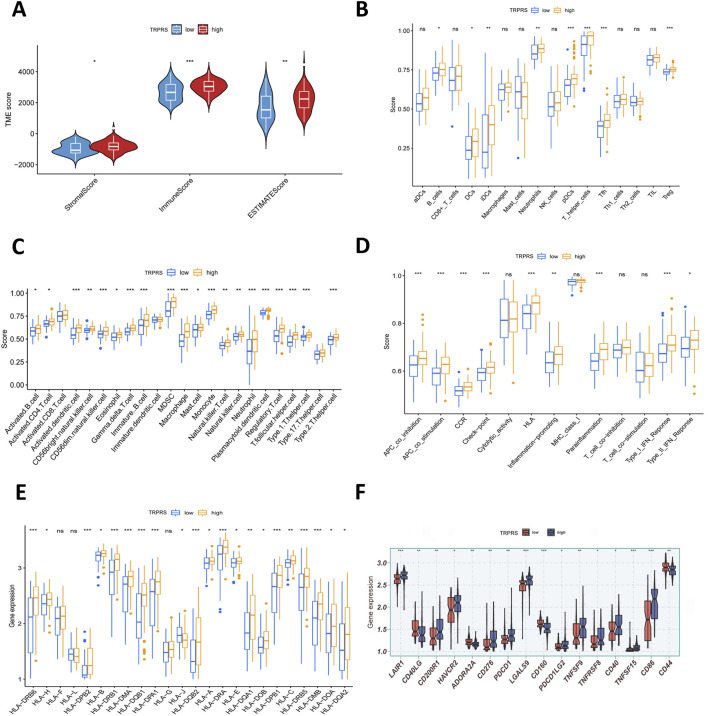
Immune microenvironment of patients with different TRPRS risk scores. **(A)** Immune, ESTIMATE, and stromal scores comparing high- and low-TRPRS groups. **(B)** Activity of immune-related pathways between high- and low-TRPRS groups. **(C,D)** TME-infiltrating cell abundance quantified by CIBERSORT and ssGSEA across groups. **(E)** Differential expression of HLA molecules in high- and low-TRPRS groups. **(F)** Differential expression of various immune checkpoints in high- and low-TRPRS groups.

GSVA was conducted to explore potential functional pathway differences between the high- and low-TRPRS groups. Although variations were observed between groups, only a subset of pathways demonstrated statistically significant enrichment, including tryptophan metabolic processes, which were more prominent in the high-TRPRS group (Figure 6A). The ESTIMATE algorithm showed that high-TRPRS patients had moderately higher immune, stromal, and total ESTIMATE scores (p < 0.05; Figure 6B). These findings suggest differences in the overall TME composition between groups, albeit with limited effect sizes (Deng et al., 2020; Jiang et al., 2021).

To further characterize immune features, immune cell infiltration was estimated using ssGSEA. Several immune cell populations, such as activated B cells, dendritic cells, myeloid-derived suppressor cells, NK cells, and Th1 cells, exhibited higher infiltration trends in the high-TRPRS subgroup (Figure 6C). However, not all differences reached statistical significance, and these results should be interpreted as exploratory. Analyses of immune-related functional signatures similarly revealed increased activity in several pathways, including type I/II interferon responses, APC co-stimulation and co-inhibition, immune checkpoint–related functions, and HLA pathways, within the high-TRPRS group (Figure 6D). While these patterns suggest potential immune dysregulation, they do not establish a causal relationship with the TRPRS. Gene expression analysis indicated that several antigen-presentation-associated genes showed higher expression levels in the high-TRPRS group (Figure 6E). Additionally, key immune checkpoint molecules, such as HAVCR2 and PDCD1, were positively correlated with TRPRS values (p < 0.05; Figure 6F). These associations may reflect differences in immune activation or regulatory processes. However, further experimental validation is required to elucidate their biological significance.

### 3.7 Development and validation a six-gene TRPRG signature for prognostic prediction

**FIGURE 7:**
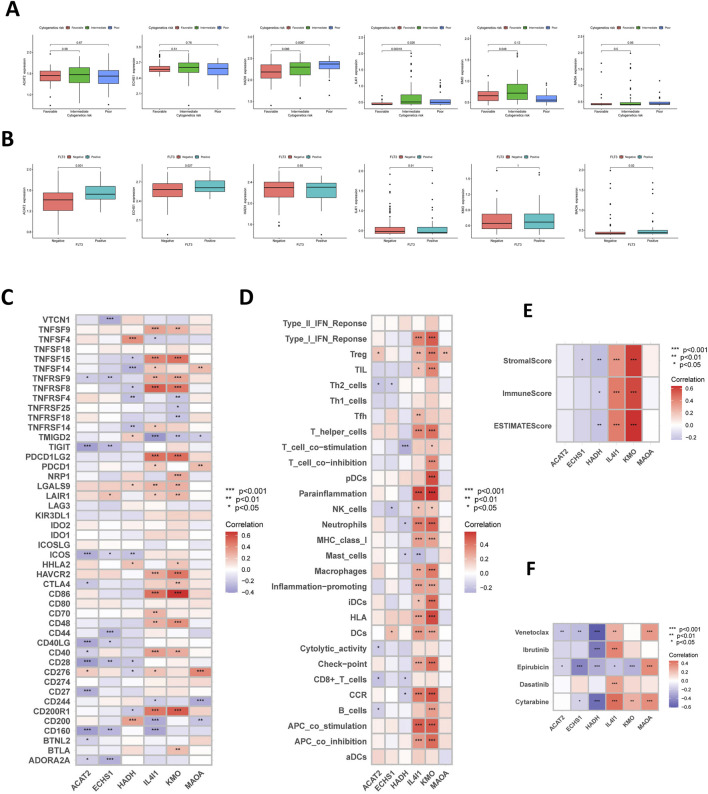
Six TRPRGs in the prognostic model: correlations with immune infiltration and drug response. **(A)** The boxplot showing the relationship of ACAT2, ECHS1, HADH, IL4I1, KMO, and MAOA expression and cytogenetic risk classification. **(B)** The boxplot depicting the correlation of ACAT2, ECHS1, HADH, IL4I1, KMO, and MAOA expression and FLT3 mutation status. **(C)** The correlation of six TRPRGs and immune checkpoints. **(D)** The relationship between six TRPRGs and immune cells. **(E)** The correlation of six TRPRGs and TME score. **(F)** The relationship between six TRPRGs and commonly used chemotherapeutic and targeted agents for AML.

The expression patterns of the six prognostic TRPRGs were evaluated in AML patients. Given the complexity of AML, driven by both intrinsic and extrinsic factors, the TRPRG risk model was analyzed alongside key clinical parameters, FAB classification, cytogenetic risk, age, and WBC count, to evaluate its prognostic relevance, with further details provided in Table 1. The findings revealed that HADH and IL4I1 expression levels were significantly associated with cytogenetic classification (Figure 7A), while ACAT2, ECHS1, and MAOA expression correlated with the presence of FLT3 mutations in AML samples (Figure 7B). Subsequently, we next examined the relationship of the six prognostic tryptophan metabolism-related genes with the TME score. Among these, ECHS1 and HADH exhibited significant negative correlations with TME activity, suggesting a possible connection to immune modulation in AML (Figure 7E). Furthermore, relationships between the six prognostic TRPRGs and key immune checkpoints were evaluated. IL4I1, KMO, and HADH demonstrated positive correlations with most checkpoints (PDCD1LG2, CD200R1, CD86, TNFSF15), whereas ACAT2 and ECHS1 exhibited inverse correlations, indicating differential roles of these genes in modulating the AML immune microenvironment (Figure 7C). Correlation analyses of the six prognostic genes with immune cell infiltration demonstrated that IL4I1, KMO, and HADH exhibited positive correlations with the majority of tumor-infiltrating immune cells, while HADH and ECHS1 showed significant negative associations with these immune populations (Figure 7D). We further evaluated correlations between the six TRPRGs and sensitivity to standard chemotherapeutics and targeted agents in AML (Figure 7F). The finding revealed differential associations between TRPRG expression and drug response: MAOA positively correlated with venetoclax sensitivity, whereas ECHS1 showed a negative correlation with epirubicin response. In contrast, HADH showed no association, indicating that these prognostic TRPRGs may modulate therapeutic outcomes in AML and serve as potential biomarkers for treatment stratification.

**TABLE 1:** Prognostic factors associated with the TRPRS in TCGA-LAML patients.

Characteristics	Total	High	Low	Pvalue
Age	53.27 ± 16.33	56.59 ± 16.21	49.95 ± 15.89	0.019
WBC	34.38 ± 42.31	42.71 ± 43.81	25.92 ± 39.29	0.0225
BMblast	38.11 ± 31.29	37.83 ± 30.35	38.39 ± 32.44	0.9185
Blast	65.67 ± 23.19	67.59 ± 20.73	63.74 ± 25.42	0.3424
Gender	​	​	​	0.1625
FEMALE	61 (46.21%)	26 (39.39%)	35 (53.03%)	​
MALE	71 (53.79%)	40 (60.61%)	31 (46.97%)	​
Age	​	​	​	0.0732
<=65	98 (74.24%)	44 (66.67%)	54 (81.82%)	​
>65	34 (25.76%)	22 (33.33%)	12 (18.18%)	​
FAB	​	​	​	4.00E-04
M0	12 (9.09%)	2 (3.03%)	10 (15.15%)	​
M1	32 (24.24%)	20 (30.3%)	12 (18.18%)	​
M2	32 (24.24%)	14 (21.21%)	18 (27.27%)	​
M3	14 (10.61%)	2 (3.03%)	12 (18.18%)	​
M4	27 (20.45%)	14 (21.21%)	13 (19.7%)	​
M5	12 (9.09%)	11 (16.67%)	1 (1.52%)	​
M6	2 (1.52%)	2 (3.03%)	0 (0%)	​
M7	1 (0.76%)	1 (1.52%)	0 (0%)	​
Cytogenetics risk	​	​	​	0.0127
Favorable	30 (22.73%)	8 (12.12%)	22 (33.33%)	​
Intermediate	73 (55.3%)	45 (68.18%)	28 (42.42%)	​
Poor	27 (20.45%)	12 (18.18%)	15 (22.73%)	​
Unknown	2 (1.52%)	1 (1.52%)	1 (1.52%)	​
FLT3	​	​	​	0.3501
Negative	90 (68.18%)	42 (63.64%)	48 (72.73%)	​
Positive	42 (31.82%)	24 (36.36%)	18 (27.27%)	​
NPMc	​	​	​	1
Negative	24 (18.18%)	12 (18.18%)	12 (18.18%)	​
Positive	108 (81.82%)	54 (81.82%)	54 (81.82%)	​

### 3.8 HADH and ECHS1 promote AML cells progression

**FIGURE 8:**
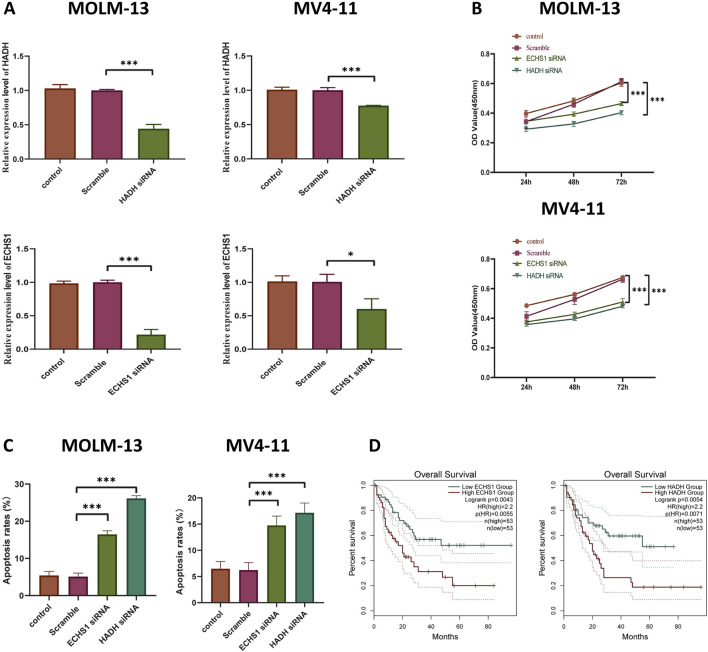
Functional roles of HADH and ECHS1 in AML progression and tryptophan metabolism. **(A)** RT-qPCR to verify HADH and ECHS1 expression in MOLM-13 cells and MV4-11 cells. **(B)** CCK8 assay in two AML cells. **(C)** Knockdown of HADH and ECHS1 accelerated apoptosis in 2 cell lines, as demonstrated by flow cytometry. **(D)** Kaplan-Meier curve analysis of HADH and ECHS1 in AML patients and normal controls.

HADH and ECHS1 were selected for experimental validation due to their strong and consistent associations with poor prognosis, higher TRPRS values, and differential expression across tryptophan-related molecular subtypes. Both genes function as key enzymes in mitochondrial fatty acid β-oxidation, a pathway integral to metabolic reprogramming in AML (Hu et al., 2022). Their significant correlations with immune checkpoint genes and TME features suggest potential functional relevance, warranting their prioritization for *in vitro* assays. To explore the roles of HADH and ECHS1 in AML, we downregulated their expressions in MV4-11 and MOLM13 cells via siRNA infection. The downregulation of HADH and ECHS1 was confirmed through RT-qPCR (Figure 8A). Comparatively, both the non-targeting siRNA (si-NC) and mock control groups indicated significant knockdown efficiency, affirming the specificity of gene silencing. The knockdown of HADH and ECHS1 markedly inhibited the proliferation of MV4-11 and MOLM13 cells (Figure 8B). Flow cytometry assessments indicated that silencing HADH and ECHS1 notably enhanced the apoptotic rates in AML cells (Figure 8C). Furthermore, increased expression levels of HADH and ECHS1 were associated with poorer survival outcomes in AML patients (Figure 8D), indicating that these genes may function as potential oncogenic activators in this malignancy.

## 4 Discussion

Tryptophan metabolism is essential in substance catabolism, and its disruption has been recognized as a significant factor in disease development and tumorigenesis (Jones et al., 2018; Kreitz et al., 2019). Contemporary studies have demonstrated that tryptophan metabolism may be associated with the TME and immunogenicity of tumors (Zhou et al., 2022; Yang et al., 2023). However, the overall impact of tryptophan metabolism—related genes and their association with tumor immune infiltration in AML remain largely unexplored. Thus, this study characterized the molecular features of TRPRGs to elucidate their relationship with clinical outcomes and the tumor immune microenvironment. Furthermore, we examined the association of TRPRG expression with overall survival in AML, resulting in the identification of seven crucial genes. These genes were used to construct the TRPRS, designed to estimate clinical outcomes and guide therapeutic strategies. Moreover, we developed a hybrid nomogram integrating clinical parameters with a quantitative scoring system to predict overall survival in AML patients. Functional annotation analyses revealed significant enrichment of tryptophan metabolism–related pathways and immune-associated hallmarks in the high-TRPRS group, suggesting that elevated tryptophan metabolism may modulate the function and phenotype of tumor-infiltrating immune cells, consistent with previous reports (Seo and Kwon, 2023).

The modulation of immune responses plays a pivotal role in AML pathogenesis and progression, as variations in the abundance and composition of tumor-infiltrating immune cells can significantly affect disease outcomes, prognostic assessment, and responses to immunotherapy (Yao et al., 2021). In this study, increased levels of monocytes and macrophages identified elevated in the high-TRPRS subgroup, reflecting a state of immune suppression. This immunosuppressive microenvironment likely mediates adverse outcomes in high-TRPRS cases and may compromise the efficacy of immunotherapeutic interventions. Nonetheless, the immunotherapy relevance of the TRPRS was inferred indirectly from computational indicators rather than validated in AML patients undergoing immune-based therapies. Therefore, the results should be interpreted cautiously, with future studies required to confirm their predictive value in clinical settings.

The pronounced disparities between the low- and high-TRPRS cohorts prompted further examination of group-specific gene expression profiles, revealing that HADH and ECHS1 play critical roles in the pathogenesis and advancement of AML. Furthermore, elevated expression of HADH and ECHS1 has been significantly associated with cancer progression and adverse clinical outcomes across various malignancies (Du et al., 2021; Yu et al., 2023), which aligns with the results of this study. Consequently, HADH and ECHS1 represent promising therapeutic targets in AML.

Consistent with established prognostic frameworks in AML, age and cytogenetic risk were also significant predictors of overall survival in our cohort. In the univariate model, both advanced age (hazard ratio [HR] = 2.67; 95% confidence interval [CI]: 1.70–4.19; p < 0.001) and adverse cytogenetics (HR = 3.15; 95% CI: 1.56–6.34; p = 0.001) were strongly associated with poorer outcomes. Importantly, after adjusting for major clinical covariates, the TRPRS remained an independent prognostic factor, whereas age (HR = 2.29; 95% CI: 1.45–3.64; p < 0.001) and cytogenetic risk (HR = 2.56; 95% CI: 1.25–5.23; p = 0.010) continued to show significant effects. These findings underscore the incremental predictive value of the TRPRS beyond conventional risk determinants and highlight its potential for refining current stratification systems. Our TRPRG signature not only served as an independent prognostic factor but also correlated with key clinical parameters. The association between the high-TRPRS group and adverse clinical features, such as advanced age and adverse cytogenetics, underscores its potential utility in refining existing risk stratification systems.

This study has several limitations. First, the TRPRS was developed using the TCGA-LAML dataset and validated across two independent GEO cohorts, which may restrict the generalizability of the findings. Although consistent results were observed across datasets, additional external cohorts are needed to further confirm the robustness of the model. Second, functional validation experiments were performed in only two AML cell lines, and rescue assays and *in vivo* studies were not conducted, limiting the mechanistic depth of the findings. Future research incorporating multiple AML models and complementary experimental approaches is required to further elucidate the biological roles of TRPRGs. Third, the immunotherapy relevance of TRPRS was inferred indirectly through immune profiling, checkpoint analysis, and TIDE prediction, lacking validation in clinical immunotherapy-treated AML cohorts. This limitation undermines the strength of conclusions regarding therapeutic applicability, suggesting the necessity for future studies incorporating real-world immunotherapy data.

## 5 Conclusion

This study established and validated a novel prognostic indicator derived from tryptophan metabolism-related genes in AML, serving as a valuable resource for enhancing patient outcomes and guiding individualized therapeutic decisions. Furthermore, this study conducted a comprehensive exploration of signaling pathways, chemosensitivity patterns, and immune infiltration within the framework of this risk model, contributing foundational insights for future targeted treatment approaches in AML.

## Data Availability

The datasets presented in this study can be found in online repositories. The names of the repository/repositories and accession number(s) can be found in the article/Supplementary Material.
