# Supplementary figures and images for: Prediction of prognosis and immunotherapy response of tryptophan metabolism genes in acute myeloid leukemia
Source: Front Pharmacol. 2026 Jan 7;16:1714246. doi: 10.3389/fphar.2025.1714246 (PMC12819808; doi:10.3389/fphar.2025.1714246)

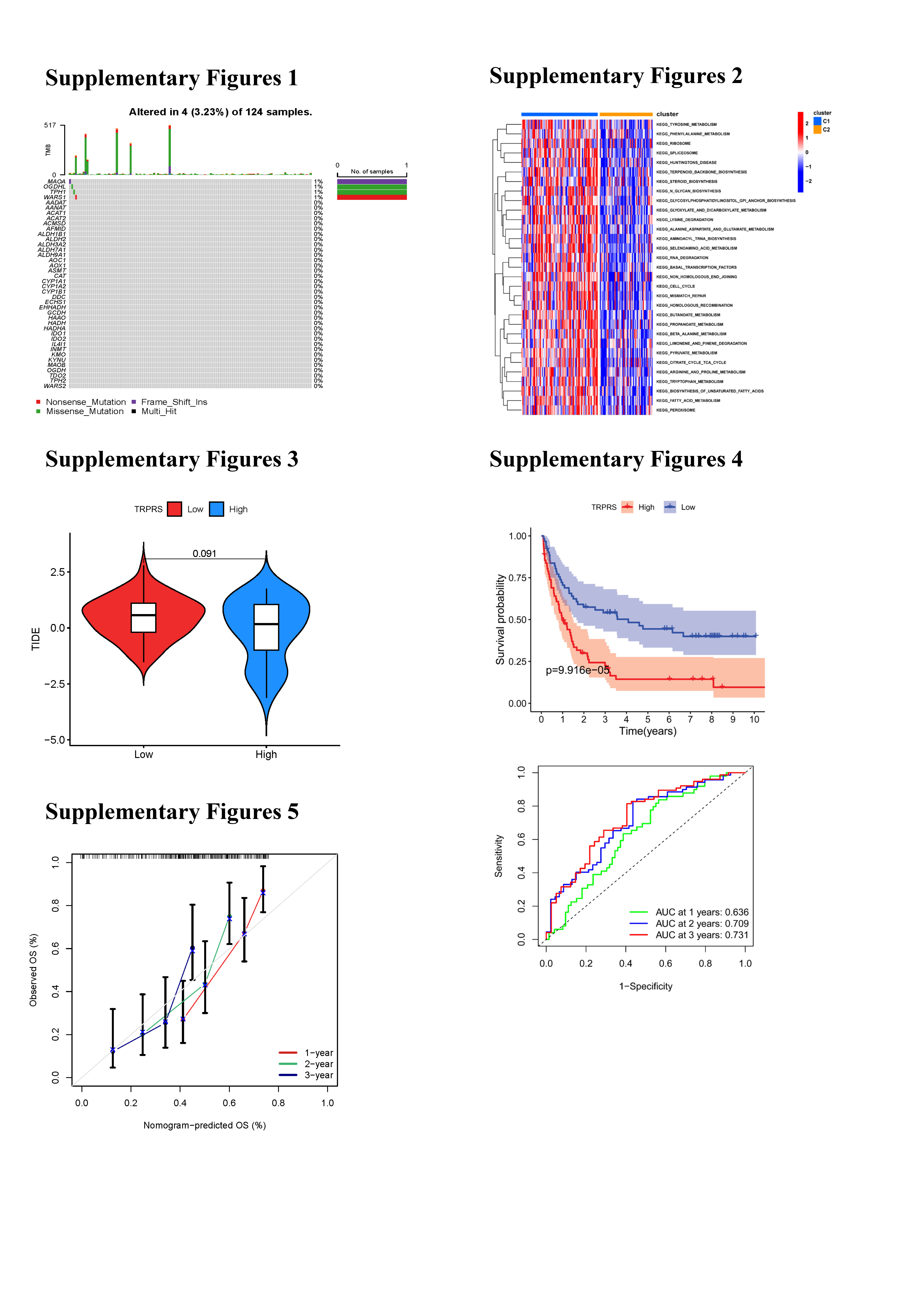

Supplement: Supplementary file 1 [file Image1.jpeg]
